# Association between body mass index and fatty liver risk: A dose-response analysis

**DOI:** 10.1038/s41598-018-33419-6

**Published:** 2018-10-15

**Authors:** Rui Fan, Jufang Wang, Jinman Du

**Affiliations:** 1 Medical quality management office, Ningbo Medical Center Lihuili Eastern Hospital, Ningbo, Zhejiang, 315040 China; 2 Medical quality management office, Taipei Medical University Ningbo Medical Center, Ningbo, Zhejiang, 315040 China; 3 Physical examination center, Ningbo Medical Center Lihuili Eastern Hospital, Ningbo, Zhejiang, 315040 China

## Abstract

Body mass index (BMI) is associated with fatty liver risk, however, the dose-response relationship between continuous BMI changes and fatty liver risk has not been clearly defined. In this study, a cross-sectional study was conducted and a total of 3202 individuals were included. Unconditional logistic regression and restricted cubic spline model were used to analyze the dose-response association of BMI with fatty liver risk. After adjusting for confounding factors (age, gender, hypertension, total cholesterol, triglycerides, glucose, high-density lipoprotein, low-density lipoprotein, uric acid, homocysteine, creatinine, aspartate aminotransferase and alanine transaminase), overweight (OR = 3.55, 95% CI: 2.49–5.06, *P* = 2.79 × 10^−12^), obesity (OR = 7.59, 95% CI: 4.91–11.71, *P* = 6.56 × 10^−20^) were significantly related to fatty liver risk. Stratified by gender (male/female), age (<50 years/≥50 years), prevalence of hypertension (yes/no), the above association was still significant (*P* = 0.004 or lower). In dose-response analysis, BMI was statistically significantly associated with fatty liver risk in a nonlinear fashion (approximately J-shaped fashion, *P*_*nonlinearity*_ = 1.71 × 10^−4^ or lower) in the total population and all subgroups mentioned above. Findings from this dose-response analysis suggest that higher BMI (overweight/obesity) is an independent, dose-dependent risk factor for fatty liver, and prevention of fatty liver focusing on continuous changes in BMI should be noted.

## Introduction

Fatty liver is a worldwide disease, the prevalence of fatty liver is increasing with great changes in lifestyle. Studies showed that the mean prevalence of fatty liver was more than 25.24% in the world^1^, 16.73% in China and was predicted to reach 20.21% by 2020^2^, which seriously affects people’s health.

By analyzing fatty liver risk factors, studies found that body mass index (BMI), one of the most classical epidemiological indexes assessing obesity, was associated with the risk of fatty liver^3–6^. Compared with normal BMI, the risk of fatty liver was approximately 4.1 to 14-fold increased in higher BMI^4,5^. Although a number of studies reported the association between BMI and fatty liver disease, previous studies were mostly limited to dividing the BMI into categorical variables (underweight, normal, overweight and obesity)^4–6^, which could not show the dose-response relationship.

As one of the analytical methods describing the dose-response relationship between continuous exposure and outcome, the restricted cubic combines a spline function with a generalized linear model such as logistic regression, and can visually present the effect of small changes of independent variables on the OR value of the corresponding variable by the form of a continuous curve^7,8^. With this method, previous studies found a dose-response relationship between BMI and diabetes and hyperlipidemia^9,10^. However, the dose-response relationship between BMI and fatty liver risk is unclear. Therefore, we aimed to explore the dose-response relationship between them using the restricted cubic spline model.

## Results

### Characteristics of the Study Population

A total of 368 fatty liver cases (317 males) and 2835 non-fatty liver subjects (1744 males) were recruited in the current study. The mean age was (42.43 ± 10.29) years in fatty liver subjects, (40.73 ± 10.48) years in non-fatty liver subjects. Compared with non-fatty liver subjects, the participants with fatty liver had higher BMI values (mean ± standard deviation: 26.82 ± 2.89 kg/m^2^ vs. 22.89 ± 2.86 kg/m^2^, *t* = −24.71, *P* = 1.76 × 10^−123^). The distribution of key variables considered in this study for fatty liver and non-fatty liver is shown in Table 1. Significant differences were found in age, gender, hypertension, total cholesterol, triglycerides, glucose, HDL, LDL, uric acid, Hcy, creatinine, AST, ALT. (*P* = 0.023 or lower).

**Table 1:** Characteristics of the study population (n = 3203).

Characteristics	Non-fatty liver (n = 2835)	Fatty liver (n = 368)	Statistics values	*P*
Age (years), Mean (SD)	40.73(10.48)	42.43(10.29)	−2.94^a^	0.003
Gender (M/F)	1744/1091	317/51	86.09^b^	1.72 × 10^−20^
Hypertension (Y/N)	407/2428	107/261	52.39^b^	4.56 × 10^−13^
BMI (kg/m^2^)				
As a continuous variable, Mean (SD)	22.89(2.86)	26.82(2.89)	−24.71^a^	1.76 × 10^−123^
As a categorical variable			507.51^b^	1.13 × 10^−109^
<18.5 (n)	141	0		
18.5–23.9 (n)	1725	53		
24–27.9 (n)	834	198		
≥28 (n)	135	117		
Total cholesterol (mmol/L), Mean (SD)	4.74(0.88)	5.06(0.90)	−6.54^a^	7.31 × 10^−11^
Triglycerides (mmol/L), Median (IQR)	1.13(0.84)	2.02(1.34)	−12.98^c^	1.55 × 10^−32^
Glucose (mmol/L), Median (IQR)	4.99(0.61)	5.23(0.90)	−6.96^c^	1.41 × 10^−11^
HDL (mmol/L), Mean (SD)	1.48(0.33)	1.26(0.25)	15.61^a^	6.67 × 10^−46^
LDL (mmol/L), Mean (SD)	2.87(0.71)	3.25(0.71)	−9.56^a^	2.29 × 10^−21^
Uric acid (mmol/L), Mean (SD)	340.11(86.65)	418.65(91.27)	−16.26^a^	3.58 × 10^−57^
Hcy (µmol/L), Median (IQR)	10.70(4.20)	11.50(4.40)	−2.27^c^	0.023
Creatinine (µmol/L), Mean (SD)	67.96(14.09)	72.55(12.40)	−6.57^a^	1.28 × 10^−10^
AST (U/L), Median (IQR)	20.00(7.00)	27.00(12.00)	−12.13^c^	4.16 × 10^−29^
ALT (U/L), Median (IQR)	19.00(14.00)	42.00(30.75)	−14.77^c^	1.51 × 10^−39^

^a^*T*-test; ^b^Pearson chi-square test; ^c^Mann-Whitney U test; SD: standard deviation; IQR: interquartile range.

### Logistic Regression Analyses for Investigation of the Association between BMI and Fatty liver

As shown in Table 2, BMI levels were significantly associated with fatty liver in total subjects and in the subgroups stratified by gender (male/female), age (<50 years/≥50 years), prevalence of hypertension (yes/no) via unadjusted logistic regression and age- and gender-adjusted logistic regression (*P* = 3.10 × 10^−6^ or lower). After adjustment for age, gender, hypertension, total cholesterol, triglycerides, glucose, HDL, LDL, uric acid, Hcy, creatinine, AST, ALT, overweight (total: OR = 3.55, 95% CI: 2.49–5.06, *P* = 2.79 × 10^−12^; male: OR = 3.41, 95% CI: 2.29–5.08, *P* = 1.57 × 10^−9^; female: OR = 3.55, 95% CI: 1.52–8.25, *P* = 0.003; < 50 years: OR = 4.15, 95% CI: 2.67–6.45, *P* = 2.63 × 10^−10^; ≥50 years: OR = 2.74, 95% CI: 1.48–5.07, *P* = 0.001; hypertension: OR = 3.66, 95% CI: 1.60–8.39, *P* = 0.002; non-hypertension: OR = 3.37, 95% CI: 2.27–5.00, *P* = 1.69 × 10^−9^, respectively), and obesity (total: OR = 7.59, 95% CI: 4.91–11.71, *P* = 6.56 × 10^−20^; male: OR = 7.04, 95% CI: 4.33–11.45, *P* = 3.33 × 10^−15^; female: OR = 14.80, 95% CI: 5.00–43.80, *P* = 1.12 × 10^−6^; <50 years: OR = 9.62, 95% CI: 5.76–16.07, *P* = 5.40 × 10^−18^; ≥50 years: OR = 3.82, 95% CI: 1.54–9.47, *P* = 0.004; hypertension: OR = 6.40, 95% CI: 2.58–15.88, *P* = 6.31 × 10^−5^; non-hypertension: OR = 8.21, 95% CI: 4.94–13.62, *P* = 3.95 × 10^−16^, respectively) were independently associated with increased risks of fatty liver.

**Table 2:** Logistic regression analyses for investigation of the association between BMI and fatty liver.

BMI (kg/m^2^)	Model 1		Model 2		Model 3	
	OR (95% CI)	*P*	OR (95% CI)	*P*	OR (95% CI)	*P*
Total						
<18.5	0	0.996	0	0.996	0	0.996
18.5–23.9	Reference		Reference		Reference	
24–27.9	7.28(5.64–10.58)	2.91 × 10^−37^	6.45(4.67–8.92)	1.61 × 10^−29^	3.55(2.49–5.06)	2.79 × 10^−12^
≥28	28.21(19.51–40.79)	1.75 × 10^−70^	23.96(16.47–34.86)	6.59 × 10^−62^	7.59(4.91–11.71)	6.56 × 10^−20^
Gender						
Male						
<18.5	0	0.998	0	0.998	0	0.998
18.5–23.9	Reference		Reference		Reference	
24–27.9	6.01(4.19–8.63)	1.94 × 10^−22^	6.01(4.19–8.63)	2.73 × 10^−22^	3.41(2.29–5.08)	1.57 × 10^−9^
≥28	21.44(14.11–32.58)	8.94 × 10^−47^	21.44(14.11–32.58)	8.95 × 10^−47^	7.04(4.33–11.45)	3.33 × 10^−15^
Female						
<18.5	0	0.997	0	0.997	0	0.997
18.5–23.9	Reference		Reference		Reference	
24–27.9	8.48(4.25–16.95)	1.42 × 10^−9^	7.34(3.62–14.88)	3.18 × 10^−8^	3.55(1.52–8.25)	0.003
≥28	36.38(15.81–83.76)	2.96 × 10^−17^	30.11(12.85–70.56)	4.63 × 10^–15^	14.80(5.00–43.80)	1.12 × 10^−6^
Age						
<50 years						
<18.5	0	0.996	0	0.996	0	0.996
18.5–23.9	Reference		Reference		Reference	
24–27.9	9.65(6.521–14.28)	8.12 × 10^−30^	7.90(5.28–11.84)	1.09 × 10^−23^	4.15(2.67–6.45)	2.63 × 10^−10^
≥28	39.06(25.10–60.80)	2.76 × 10^−59^	31.89(20.72–50.15)	9.58 × 10^−51^	9.62(5.76–16.07)	5.40 × 10^−18^
≥50 years						
<18.5	0	0.998	0	0.998	0	0.998
18.5–23.9	Reference		Reference		Reference	
24–27.9	4.38(2.57–7.46)	5.49 × 10^−8^	4.03(2.35–6.91)	4.09 × 10^−7^	2.74(1.48–5.07)	0.001
≥28	11.07(5.38–22.75)	6.24 × 10^−11^	10.64(5.16–21.94)	1.53 × 10^−10^	3.82(1.54–9.47)	0.004
Hypertension						
Yes						
<18.5	0	0.999	0	0.999	0	0.999
18.5–23.9	Reference		Reference		Reference	
24–27.9	5.71(2.74–11.91)	3.41 × 10^−6^	6.02(2.83–12.80)	3.10 × 10^−6^	3.66(1.60–8.39)	0.002
≥28	15.93(7.27–34.87)	4.42 × 10^−12^	15.92(7.19–35.28)	9.14 × 10^−12^	6.40(2.58–15.88)	6.31 × 10^−5^
No						
<18.5	0	0.996	0	0.996	0	0.996
18.5–23.9	Reference		Reference		Reference	
24–27.9	7.75(5.46–11.00)	1.95 × 10^−30^	6.15(4.29–8.81)	4.17 × 10^−23^	3.37(2.27–5.00)	1.69 × 10^−9^
≥28	31.13(20.19–48.00)	1.23 × 10^−54^	25.65(16.50–39.87)	4.23 × 10^−47^	8.21(4.94–13.62)	3.95 × 10^−16^

Model 1: unadjusted; Model 2: adjusted for age and/or gender; Model 3: adjusted for age and/or gender and/or hypertension, total cholesterol, triglycerides, glucose, HDL, LDL, uric acid, Hcy, creatinine, AST, ALT.

### Dose-Response Relationship between BMI and Fatty liver

In dose-response analysis, BMI was associated with fatty liver risk in a nonlinear fashion (approximately J-shaped fashion*, P*_*nonlinearity*_ = 1.71 × 10^−4^ or lower) with a significantly increased trend of odds ratio as per 1 kg/m^2^ increase in BMI, in the total population and all subgroups mentioned above, after adjusting for age, gender, hypertension, total cholesterol, triglycerides, glucose, HDL, LDL, uric acid, Hcy, creatinine, AST, ALT.

**Figure 1:**
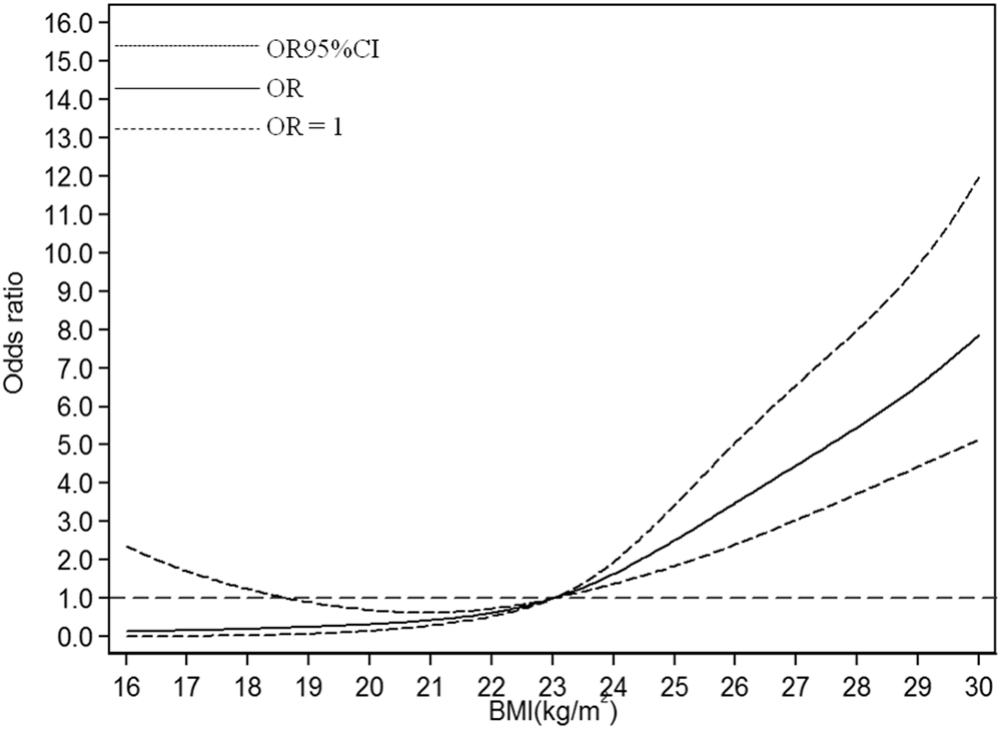
Association between BMI and fatty liver risk based on restricted cubic spline model in total population.

In the total subjects, the fitted dose-response relationship was described in Fig. 1 (*P*_*nonlinearity*_ = 1.71 × 10^−4^). When compared with the reference (BMI = 23 kg/m^2^), the ORs (95% CI) for fatty liver risks were 0.23 (0.05–1.02) for BMI at 18.6 kg/m^2^, 0.62 (0.54–0.73) for BMI at 22 kg/m^2^, 2.06 (1.60–2.66) for BMI at 24.5 kg/m^2^, and 6.09 (4.15–8.94) for BMI at 28.6 kg/m^2^, indicating a significant and progressive risk of fatty liver along with BMI increases.

**Figure 2:**
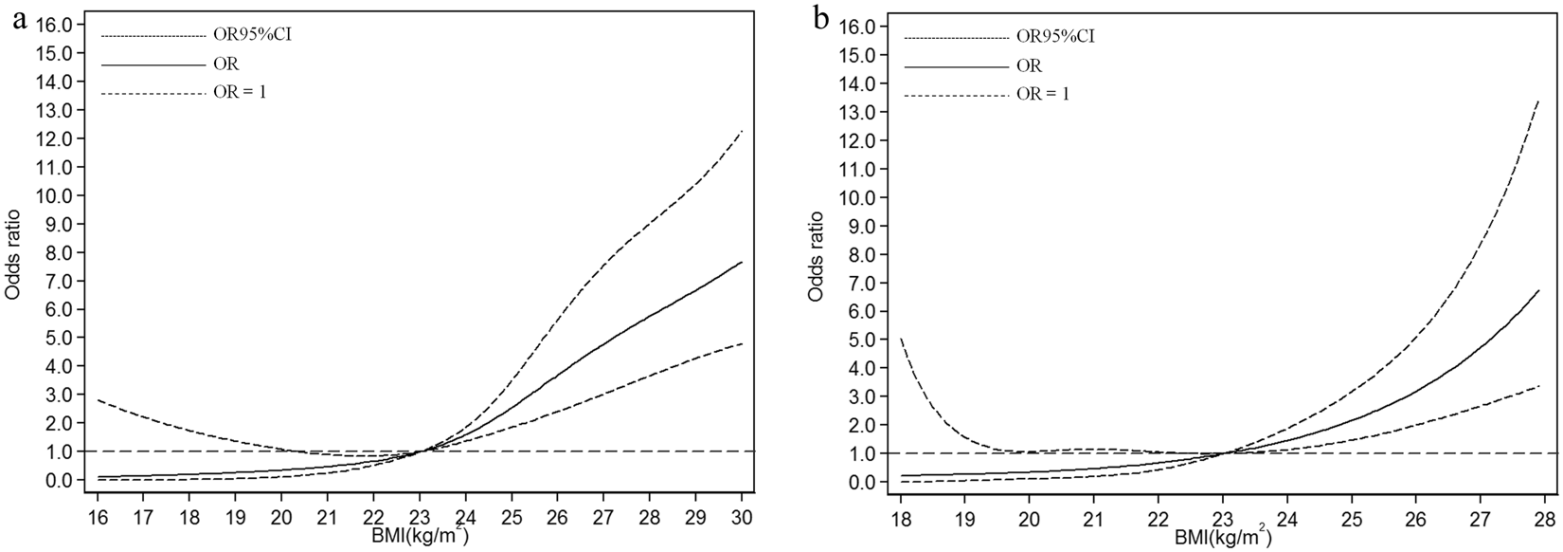
Association between BMI and fatty liver risk based on restricted cubic spline model in male and female (**a**) male; (**b**) female.

In the subgroups stratified by gender, the fitted dose-response relationships were described in Fig. 2. In males (Fig. 2a, *P*_*nonlinearity*_ = 2.62 × 10^−5^), when compared with the reference (BMI = 23 kg/m^2^), the ORs (95% CI) for fatty liver risks were 0.24 (0.04–1.51) for BMI at 18.6 kg/m^2^, 0.67 (0.53–0.85) for BMI at 22 kg/m^2^, 2.04 (1.61–2.58) for BMI at 24.5 kg/m^2^, and 6.31 (4.05–9.83) for BMI at 28.6 kg/m^2^. In females (Fig. 2b, *P*_*nonlinearity*_ = 3.23 × 10^−5^), when compared with the reference (BMI = 23 kg/m^2^), the ORs (95% CI) for fatty liver risks were 0.25 (0.03–2.35) for BMI at 18.6 kg/m^2^, 0.68 (0.44–1.05) for BMI at 22 kg/m^2^, 1.78 (1.29–2.48) for BMI at 24.5 kg/m^2^, and (3.96–19.80) for BMI at 28.6 kg/m^2^.

**Figure 3:**
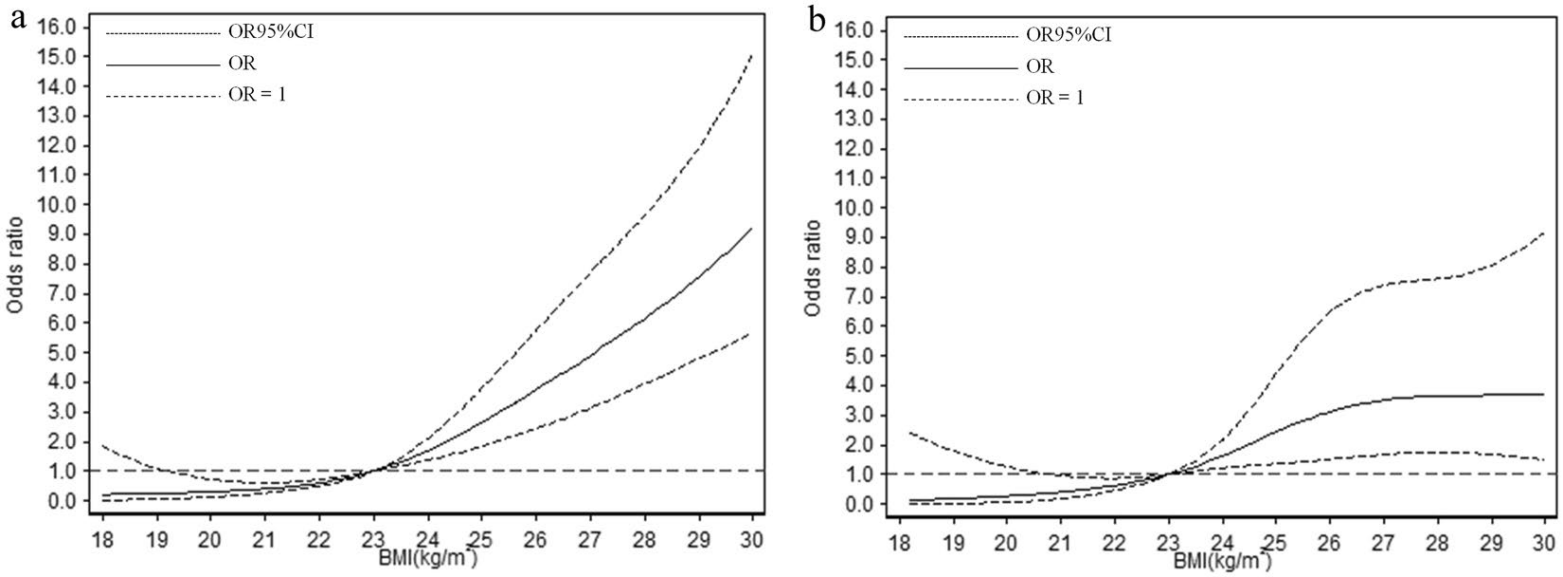
Association between BMI and fatty liver risk based on restricted cubic spline model in different age groups (**a**) <50 years; (**b**) ≥50 years.

In the subgroups stratified by age, the fitted dose-response relationships were described in Fig. 3. In the group <50 years (Fig. 3a, *P*_*nonlinearity*_ = 4.73 × 10^−6^), when compared with the reference (BMI = 23 kg/m^2^), the ORs (95% CI) for fatty liver risks were 0.23 (0.04–1.35) for BMI at 18.6 kg/m^2^, 0.60 (0.49–0.72) for BMI at 22 kg/m^2^, 2.15 (1.59–2.90) for BMI at 24.5 kg/m^2^, and 6.98 (4.45–10.95) for BMI at 28.6 kg/m^2^. In the group ≥50 years (Fig. 3b, *P*_*nonlinearity*_ = 5.14 × 10^−5^), when compared with the reference (BMI = 23 kg/m^2^), the ORs (95% CI) for fatty liver risks were 0.15 (0.01–2.07) for BMI at 18.6 kg/m^2^, 0.63 (0.46–0.87) for BMI at 22 kg/m^2^, 2.02 (1.29–3.18) for BMI at 24.5 kg/m^2^, and 3.66 (1.72–7.81) for BMI at 28.6 kg/m^2^.

**Figure 4:**
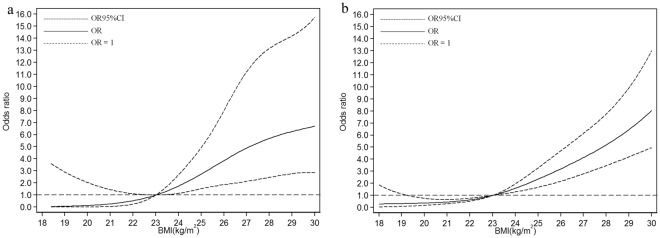
Association between BMI and fatty liver risk based on restricted cubic spline model stratified by prevalence of hypertension (**a**) yes; (**b**) no.

In the subgroups stratified by the prevalence of hypertension, the fitted dose-response relationships were described in Fig. 4. In the hypertension group (Fig. 4a, *P*_*nonlinearity*_ = 1.27 × 10^−6^), when compared with the reference (BMI = 23 kg/m^2^), the ORs (95% CI) for fatty liver risks were 0.05 (0.00–3.36) for BMI at 18.6 kg/m^2^, 0.53 (0.25–1.11) for BMI at 22 kg/m^2^, 2.22 (1.32–3.74) for BMI at 24.5 kg/m^2^, and 6.06 (2.66–13.80) for BMI at 28.6 kg/m^2^. In the non-hypertension group (Fig. 4b, *P*_*nonlinearity*_ = 3.36 × 10^−4^), when compared with the reference (BMI = 23 kg/m^2^), the ORs (95% CI) for fatty liver risks were 0.31 (0.07–1.37) for BMI at 18.6 kg/m^2^, 0.63 (0.52–0.77) for BMI at 22 kg/m^2^, 1.96 (1.48–2.60) for BMI at 24.5 kg/m^2^, and 5.91 (3.90–8.95) for BMI at 28.6 kg/m^2^.

## Discussion

The current study showed that higher BMI (overweight/obesity) was significantly associated with fatty liver risk, among which the risk of fatty liver in overweight population was 3.55 times that of the normal population (OR = 3.55), and the obese population was 7.59 times that of the normal population (OR = 7.59). Furthermore, in dose-response analysis, BMI was statistically significantly associated with fatty liver risk in a nonlinear fashion (approximate J-shaped fashion) in the total population and all subgroups stratified by gender (male/female), age (<50 years/≥50 years), and prevalence of hypertension (yes/no), indicating higher BMI is an independent, dose-dependent risk factor for fatty liver.

Similarly, a number of studies showed that elevated BMI was an independent risk factor for fatty liver^3–6^. However, previous studies were mostly limited to dividing the BMI into categorical variables (underweight, normal, overweight and obesity), and calculated the strength of its association with fatty liver disease using logistic or cox model, while ignoring the trajectory between the continuity of BMI and fatty liver risk. Moreover, according to different standards, there are distinct diagnostic points for BMI classification^11^. Artificially subdividing BMI into segments will not only lead to loss of information, but also inaccurate results. Therefore, the current study focused on describing the dose-response curve when the BMI actually underwent subtle changes so that objectively and clearly showed the correlation between them.

Based on restricted cubic spline model, we demonstrated that BMI was significantly associated with fatty liver risk in a nonlinear fashion (approximately J-shaped fashion), although there was a slight difference among the total population and different subgroups. When BMI <23 kg/m^2^, BMI had no effect on the risk of fatty liver, and when BMI >23 kg/m^2^, the risk of fatty liver disease increased significantly with a 1 kg/m^2^ increase in BMI. The possible mechanism^12,13^ is that patients with higher BMI have more adipose tissue and fatty acid flowing to the liver is increasing. Furthermore, higher BMI patients often consume a high-fat diet for a long time, which increases the absorption of exogenous fat, resulting in the increase of fatty acids and their lipidation in liver. While the synthesis of apolipoprotein B and phospholipids is relatively reduced, finally lead to the deposition of triglycerides in liver and the onset of fatty liver. In a word, higher BMI (overweight/obesity) is an independent, dose-dependent risk factor for fatty liver disease, and interventions for obesity are very necessary.

Due to the difference of BMI cut-off points between China and WHO, our study should be explained with caution. Compared with WHO BMI classification, the cutoff values are lower in China BMI classification, leading to the number of subjects with higher BMI (overweight/obesity) increases and the association between BMI and fatty liver risk varies accordingly. Therefore, the findings in the present study may not be directly applicable to white or European population. In addition, although BMI is one of the most classical epidemiological indexes assessing obesity, it does not allow a more specific analysis of composition (i.e. lean and fat masses), which means the subjects with high BMI may not be obese, and the subjects with normal BMI may be obese. Thus, the association between BMI and fatty liver risk may not be equivalent to the association between obesity and fatty liver. Ongoing studies should be conducted to confirm our findings.

To our knowledge, this is the first study reporting the non-linear dose-response relationship using a restricted cubic spline model to combine quantitative data with the occurrence of outcomes. However, a few limitations should be considered. First, the causal association between BMI and fatty liver risk is not certain because of the cross-sectional design. Second, although we tried our best to control the confounding factors, the lack of smoking, drinking and other potential confounding factors may affect the conclusion more or less. Due to the lack of drinking data, we did not subdivide fatty liver into alcoholic fatty liver and non-alcoholic fatty liver, although approximately 90% of fatty liver cases appeared to be nonalcoholic^14^. Third, fatty liver diagnosed by ultrasonography may provide an incorrect diagnosis compared with liver biopsy, although ultrasonography has been validated for diagnosing fatty liver^15^. Fourth, although the sample size was relatively large and more information was gotten through subgroup analyses stratified by gender (male/female), age (<50 years/≥50 years), and prevalence of hypertension (yes/no), subgroup analyses might be statistically inefficient due to the loss of power. Therefore, a prospective cohort study with large sample size is expected to be conducted and the data on drinking and other important factors should be collected to analyze the relationship between continuous changes in BMI and the risk of fatty liver.

In summary, our findings suggest that higher BMI (overweight/obesity) is an independent, dose-dependent risk factor for fatty liver, and prevention of fatty liver focusing on continuous changes in BMI should be noted.

## Methods

### Sample collection

We performed a cross-sectional study using data obtained from Ningbo Medical Center LiHuili Eastern Hospital in Ningbo City, Zhejiang, China. A total of 3203 individuals (2061 males, 1142 females) who visited the hospital from January 2017 to December 2017 were recruited. Subjects who were found to have viral, medical, autoimmune hepatitis, schistosomiasis liver disease, kidney disease or other serious diseases were excluded. Subjects were categorized as fatty liver according to the presence of an ultrasonographic pattern consistent with ‘bright liver’ (brightness and posterior attenuation) with stronger echoes in the hepatic parenchyma than in the renal parenchyma, vessel blurring, and narrowing of the lumen of the hepatic veins in the absence of findings suggestive of other chronic liver disease^16,17^. The study protocol was approved by the ethics committee of Ningbo Medical Center LiHuili Eastern Hospital, all methods were carried out in accordance with approved guidelines, and written informed consent was obtained.

### Data Collection and Measurements

The sociodemographic characteristics including region, age, gender, occupation were collected. In the light of the standard protocols and techniques, the participants went through anthropometric examinations including height, weight by a trained certified research practitioner. They were measured for height after taking shoes off. BMI was described as a person’s weight in kilograms divided by the square of his/her height in meters (kg/m^2^). Because of the associations between BMI, percentage of body fat, and body fat distribution differ across populations, Chinese population has lower BMI but higher percentage of body fat than white or European population^11,18^. At the same BMI level, Chinese people are at higher risk of developing chronic diseases such as type 2 diabetes and cardiovascular disease^11,18^. Therefore, the obesity categories recommended by the Chinese Ministry of Health^19^ were adopted in this study: BMI <18.5 refers to underweight, 18.5< BMI <23.9 refers to healthy weight, 24.0< BMI <27.9 refers to overweight, and BMI >28.0 refers to obese. A calibrated mercury sphygmomanometer with an adult-sized cuff was applied to assess blood pressure based on standard protocols of the American Heart Association^20^. Blood pressure was measured in the supine position twice ≥10 min apart by different trained technicians. Hypertensive patients were identified according to the ‘gold standard’^21^. All the hypertensive patients had at least three consecutive records of diastolic blood pressure (DBP) >90 mmHg and/or systolic blood pressure (SBP) >140 mmHg, or had received antihypertensive medication for >3 months^21^.

Fasting venous blood samples of each participant were extracted by venipuncture for measuring the levels of blood lipids, including total cholesterol (TC), triglycerides (TG), glucose, high-density lipoprotein (HDL), low-density lipoprotein (LDL), uric acid, homocysteine (Hcy), creatinine, aspartate aminotransferase (AST) and alanine transaminase (ALT). Blood lipids were measured using the ADVIA2400 automated biochemistry analyzer (Siemens AG, Munich, Germany) in a core laboratory with a standard protocol.

### Statistical analysis

Data were analyzed in PASW Statistics 19.0 (SPSS, Inc., Somers, NY, USA). Kolmogorov-Smirnov test was applied to test for normality and decide on the use of parametric or non-parametric tests. Continuous variables including age, BMI, total cholesterol, HDL, LDL, uric acid were presented as mean and standard deviation (SD), compared by *t*-test. Triglycerides, glucose, Hcy, AST and ALT were presented as median and interquartile range (IQR), compared by nonparametric test (Mann-Whitney U test). Categorical variables including gender, hypertension (yes/no), BMI (as a categorical variable) were compared by Pearson chi-square test. Logistic regression model was applied to analyze the correlations of BMI with fatty liver. Previous studies^22,23^ reported that male, aging (older than 50 years), hypertension were the risk factors for fatty liver, therefore, subgroup analyses stratified by gender (male/female), age (<50 years/≥50 years), and prevalence of hypertension (yes/no) were conducted. Moreover, the restricted cubic spline method was used to detect the possible nonlinear dependency of the relationship between the risk of fatty liver and BMI levels, using 4 knots at prespecified locations based on the percentiles of the distribution of BMI, the 5th, 25th, 75th, and 95th percentiles^24^. Restricted cubic spline was performed by the Stata software (version 12.0, Stata Press, College Station, TX, USA). All statistical tests were two-tailed and *P* < 0.05 was considered statistically significant.

## Data Availability

The datasets generated and analyzed during the current study are available from the corresponding authors on reasonable request.
